# Vocal Cord Actinomycosis Mimicking a Laryngeal Tumor

**DOI:** 10.1155/2013/361986

**Published:** 2013-03-21

**Authors:** Keisuke Yoshihama, Yasumasa Kato, Yuh Baba

**Affiliations:** ^1^Department of Otolaryngology, Nasu Red Cross Hospital, 1081-4 Nakatahara, Otawara City, Tochigi 324-8686, Japan; ^2^Department of Oral Function and Molecular Biology, Ohu University, 31-1 Misumido, Tomita-machi, Koriyama City, Fukushima 963-8611, Japan; ^3^Department of Otolaryngology, Keio University, 35 Shinano-machi, Shinjuku, Tokyo 160-8582, Japan

## Abstract

Laryngeal carcinoma and laryngeal papilloma are the most commonly encountered tumorous lesions in the larynx. Herein, we report a case of the mass arising from the left vocal cord in a 49-year-old Japanese man. Endoscopic examination suggested that the mass is a tumor such as carcinoma and papilloma. Pathological examination showed that the specimen demonstrated actinomycosis in the left vocal cord. Although vocal cord actinomycosis is extremely rare, the otolaryngologist should recognize this condition during the inspection of the larynx.

## 1. Introduction

Laryngeal carcinoma and laryngeal papilloma are the most commonly encountered tumorous lesions in the larynx. Actinomycosis is a disease that is mainly caused by *Actinomyces israelii*. *Actinomyces israelii* is an anaerobic, Gram-positive organism that is normally present in the oral cavity. Clinical manifestation mainly occurs in one of three forms: cervicofacial, abdominal-pelvic, or pulmonary actinomycosis. However, the actinomycosis of the larynx is very rare. Here, we report a case of primary vocal cord actinomycosis mimicking a laryngeal tumor.

## 2. Case Report

**Figure 1:**
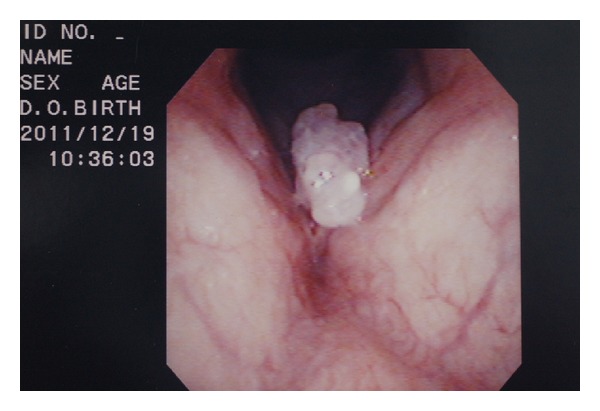
A mass of surface irregularity in the anterior one-third part of the left vocal cord.

**Figure 2:**
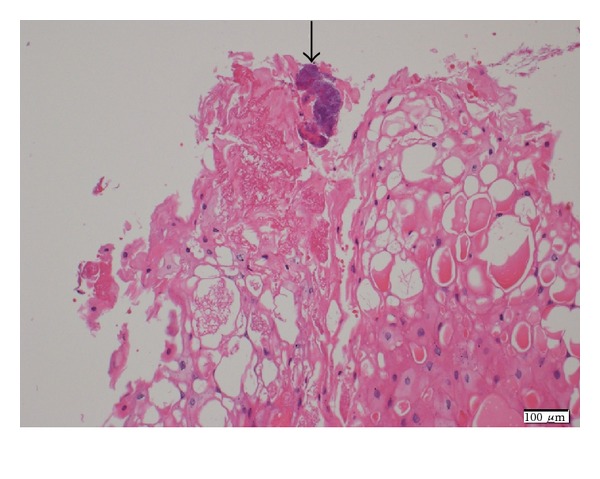
Microscopically, specimen demonstrated vocal cord actinomycosis (hematoxylin and eosin).

Approximately one year ago, a 49-year-old Japanese man came for treatment with the complaint of his hoarseness of voice for 2 years. He had no fever, odynophagia, weight loss, or dysphagia. There was no history of any operation in head and neck, and he had diabetes mellitus and dental caries. Laryngoscopy showed a mass of surface irregularity in the anterior one-third part of the left vocal cord (Figure 1). The rest of larynx and hypopharynx were normal. Examination of the neck revealed no lymphadenopathy. Laboratory investigation including complete hemogram, erythrocyte sedimentation rate, renal function tests, and liver function tests was within normal limits. The value of HbA1c was 6.1. Serological tests for HIV, HbsAg, and HCV RNA were negative. His chest roentgenogram was normal. The patient underwent direct laryngoscopy, and the mass was excised from the left vocal cord. In histopathological examination, the biopsy material revealed actinomycosis with vocal nodule (Figure 2). An infectious disease consultation was obtained, and the patient started treatment with amoxicillin-clavulanate 625 mg orally three times a day for 8 weeks. During the 2 months of treatment, the vocal cords and other laryngeal structures were observed to be normal. 

## 3. Discussion

Actinomycosis in human involves most commonly (60~70%) the cervicofacial region and is most commonly due to the *Actinomyces israelii*. *Actinomyces israelii* is a commensal saprophyte of the normal oral flora [1–4]. Trauma, for example, tooth extraction, caries, and dental manipulation, causes disruption of the normal mucosa and predisposes to infection. Debilitating and immunosuppressing factors like diabetes, pregnancy, steroid therapy, or cancer are the other predisposing factors. Differentiating diagnosis includes carcinoma, abscess, congenital anomalies, tuberculosis, fungal diseases, and osteomyelitis. Because of its complex preparation, actinomycosis may be termed “the masquerader of the head and neck.”

Laryngeal actinomycosis is quite rare. A review of the literature reveals that actinomycosis of the larynx is often associated with an underlying history of squamous cell carcinoma of the larynx and therapeutic radiation therapy [5]. An underlying history of systemic lupus erythematosus (SLE) and immunosuppression after renal transplantation has also been reported [6]. Here, we report a case of laryngeal actinomycosis without any the above mentioned underlying diseases. Laryngeal carcinoma and laryngeal papilloma are the most commonly encountered tumorous lesions in the larynx. Although actinomycosis arising from the vocal cord is extremely rare [7], we should consider this entity in the differential diagnosis of tumor and tumor-like lesions of the vocal cord.

The pathogenesis of laryngeal actinomycosis is not clear. Why the microorganism, which is normally present in the mouth, should became pathogenic is poorly understood. Poor local hygiene, local tissue damage, diabetes mellitus, immune suppression, and malnutrition are suggested predisposing factors [8]. Therefore, we postulate that in our patient dental caries and/or diabetes mellitus may cause disruption of the normal mucosal barrier and lead to infection.

In summary, we describe an additional case of vocal cord actinomycosis in this paper.
